# Going their own way–male recreational runners and running-related injuries: A qualitative thematic analysis

**DOI:** 10.1371/journal.pone.0273401

**Published:** 2022-08-25

**Authors:** Benjamin Peterson, Angela Searle, Martin Spink, Fiona Hawke, Robin Callister, Vivienne Chuter

**Affiliations:** 1 Department of Podiatry, School of Health, Medical and Applied Sciences, CQUniversity, Rockhampton, QLD, Australia; 2 School of Health Sciences, College of Heath, Medicine and Wellbeing, University of Newcastle, Central Coast Campus, Ourimbah, NSW, Australia; 3 School of Biomedical Sciences and Pharmacy, College of Heath, Medicine and Wellbeing, University of Newcastle, Callaghan Campus, Callaghan, NSW, Australia; 4 School of Health Sciences, Western Sydney University, Campbelltown Campus, Sydney, NSW, Australia; Sheffield Hallam University, UNITED KINGDOM

## Abstract

**Objective:**

Recreational running is one of the most common physical leisure activities worldwide and is associated with high rates of running related injury (RRI). Little is known of the perceptions of male recreational runners regarding the aetiology and management of RRI.

**Design:**

Utilising an interpretive phenomenological analysis framework, qualitative data was gathered from participants via interview, and reflexive thematic analysis was used to develop insights into the experiences and perceptions of the participants in relation to RRI.

**Materials and methods:**

Two focus groups with a total of six (mean age 37.8 ± 9.5 years, 16.5 ± 13.1 years running experience) male recreational runners were used to obtain data regarding their understanding of RRI causation, prevention and management. Interviews were evaluated using a six-phase reflexive thematic analysis approach to generate and interpret themes within the data.

**Results:**

Three themes (Mind, Body and Education) were identified by the analysis as critical to RRI avoidance. Mind refers to the self-understanding and self-management of personal limits required for RRI prevention. Body reflects a degree of physical conditioning necessary for injury free running, while Education indicates an understanding of how to correctly structure a running program. When viewed together these themes can be seen to form an ‘internal locus of injury’ model which highlights the runners’ beliefs that RRI are related to their decisions regarding training and running, and that avoidance of injury lies within their personal control.

**Conclusion:**

Recreational runners rely on self-management, in preference to professional advice, to manage training loads, fitness and RRI. Health care professionals involved with this population may consider the use of online resources, a preferred option of runners, to assist runners to build their knowledge base and support their development to experienced runners.

## Introduction

Despite extensive research into the aetiology of running-related injury (RRI), the rates of RRI remain high. Although RRI are generally understood to have a multifactorial aetiology [1], the most well-established risk factors include previous injury [2] and training errors [3]. Many runners also perceive training errors to be a primary cause of RRI [4, 5], however there is no evidence to support their most frequently stated training error, inadequate stretching, as a contributor to RRI [3]. Similarly, despite inconclusive evidence of the protective effect of motion controlling footwear against pronation-induced RRI [6, 7], studies investigating runners’ perceptions of risk factors for RRI found that many of the participants name inappropriate footwear as a factor [4, 5]. In the main, past RRI research has concentrated on individual intrinsic and extrinsic factors, such as musculoskeletal factors, training technique, or running shoes [3, 8]. Furthermore, research into general sports injury prevention has mainly concentrated on incidence, aetiology and injury prevention factors such as equipment and physical training [9]. While these approaches have provided a detailed understanding of injury and prevention from a biomedical, biomechanical and physiological perspective, this has not translated into a corresponding reduction in injury occurrence [10]. An alternate research approach has been suggested, with a broader focus on the athlete as a whole rather than on a specific injury, which may provide new perspectives into sports injury and prevention [10, 11].

Qualitative research methods may assist with providing this broader research focus. These methods have been suggested as a means of better understanding the complexity of sports injuries and how a runner perceives RRI and prevention, as they may uncover new parameters for investigation that have not been explored in quantitative studies [11, 12]. For instance, a qualitative study investigating the beliefs of coaches and athletes in the Netherlands towards sports injuries revealed that psychological and social factors, in addition to more highly researched physiological and physical factors, should also be considered when developing an injury prevention program [13]. Psychological or behavioural factors, not commonly described by quantitative research but identified by the athletes themselves, include not coping with self-induced pressure or stress, not knowing when to stop and feelings of impatience with pain, injury and fatigue, while the social factors included perceived pressure from parents, an audience, team mates and coaches [13]. Other qualitative studies, in Brazil and the Netherlands, have also highlighted novel factors that runners perceive to be involved in RRI and prevention, such as ‘exceeding one’s own limits’ and a preference for autonomy and self-regulation, which suggest that injury preventative strategies may be more effective when made in conjunction with runners rather than for them [4, 14].

The clinical experiences of the authors led them to investigate the perspectives and experiences of individuals and groups of specific recreational running populations in relation to the causation, prevention, and management of RRI which may be fundamental to developing more effective strategies to reduce rates of RRI, and may provide additional opportunities for education and intervention [15]. RRI is affected by demographic, cultural and running population factors, and it is likely that these factors will also affect runner’s perceptions of RRI [2, 16, 17]. Therefore, the aim of our study is to investigate the views and perceptions of Australian male recreational runners’ understanding of influences on RRI causation, prevention and management.

## Materials and methods

### Design

Within an interpretive phenomenological framework, a qualitative study employing small focus group interviews was utilised. An experiential orientation to understanding the data was employed as it was perceived to best support the research question, in this case how a given phenomenon (RRI) was experienced and understood by the runners. Interview data were analysed using reflexive thematic analysis to develop a perspective of the runners’ meaning and concepts regarding RRI causation, prevention and management.

### Participants

Six male recreational runners were recruited via snowball sampling methods in NSW Australia during August and October 2020 (Table 1). A flyer was posted to social media which two recreational runners responded to, who then each recruited two peers to participate in a focus group. A focus group interview method was chosen as it is a time efficient option that allows the participants to interact and may provide multiple perspectives on a topic [18]. The criteria for designation as a recreational runner were having a minimum of 12 months running experience, running at least twice per week, and not competing at elite levels of competition. The study was approved by the University of Newcastle Human Research Ethics Committee (H-2018-0062) and informed written consent was obtained from each participant prior to participation in their focus group. Additional verbal consent was obtained from each participant at the start of the focus group.

**Table 1:** Characteristics of focus group participants.

Characteristic	Outcome
**Age (median, IQR)**	35.0 (14.8)
**Male gender (n, percentage)**	6 (100%)
**Running experience, years (median, IQR)**	15.0 (10.8)
**Weekly distance, km (median, IQR)**	35.0 (70.6)
**History of RRI (n, percentage)**	6 (100%)

### Data collection

Each runner participated in only one of two focus groups. Each focus group included three runners and one facilitator (BP) and lasted between 45 minutes and one hour. One focus group took place in-person and the other took place online via Zoom^TM^ due to restrictions on face-to-face research during COVID-19. The interview questions were designed to elicit information regarding their experience of RRI and any factors, such as training errors or running footwear, they believe contribute to the development and prevention of RRI (S1 File). Additionally, they were asked about past occurrence of RRI and how the injury was managed, including any health professionals they consulted. Participants were encouraged to share their own experiences, as well as their perceptions of more widely held views. All participants were referred to by pseudonyms during the interview and data analysis processes to preserve their anonymity. The interviews were recorded on a digital voice recorder (Stereo IC Recorder (ICD-PX470, SONY^TM^, Konan Minato-ku Tokyo JP) and transcribed into Word documents. The facilitator (BP) is a registered male podiatrist researching quantitative and qualitative aspects of RRI for his PhD candidature. He has experience teaching undergraduate Musculoskeletal and Sports related topics and has been a keen recreational runner for ten years.

### Data analysis

Data were analysed using the six-phase reflexive thematic analysis approach proposed by Braun and Clarke (S2 File) [19, 20]. This involved initial familiarisation with the data through the process of transcription (AS and BP) and then reading the transcripts multiple times in an active way while taking notes (AS and BP). Given the limited evidence available on this topic, the initial codes were generated (AS) using a predominantly inductive coding process, which is a data driven method that approaches the data without a pre-conceived theory or coding framework, and where participant meanings are prioritised [19]. The initial codes were identified at a semantic level, reflecting the information directly communicated by the runners, although still guided by our research question of the runners’ understanding of RRI causation, prevention and management. Codes and supporting quotes were extracted to an Excel spreadsheet to allow for sorting and categorisation. Initial themes were generated (AS and BP) in an iterative process that examined codes for commonalities and moved codes in and out of categories, until a pattern of codes and themes could be established. The themes were not solely identified at a semantic level and consideration was given to possible underlying concepts not directly articulated by the runners. As the authors have front line clinical backgrounds and an interest in working with clients to improve their health and activity levels, this approach was taken to allow specific clinical recommendations to be developed, based on the runners’ experiences. The themes were then reviewed (AS and BP) to confirm the central idea or meaning of the themes captured. Specific quotations from the participants, that supported and explained the themes, were extracted to assist the discussion. Finally, the themes were defined and named (AS and BP). The person undertaking the coding (AS) is a researcher and clinician in Allied Health with an interest in assisting clients to improved health outcomes. She investigated ankle range of motion, stretching and plantar pressures for her Doctorate and has been a regular recreational runner for 30 years.

## Findings

**Fig 1:**
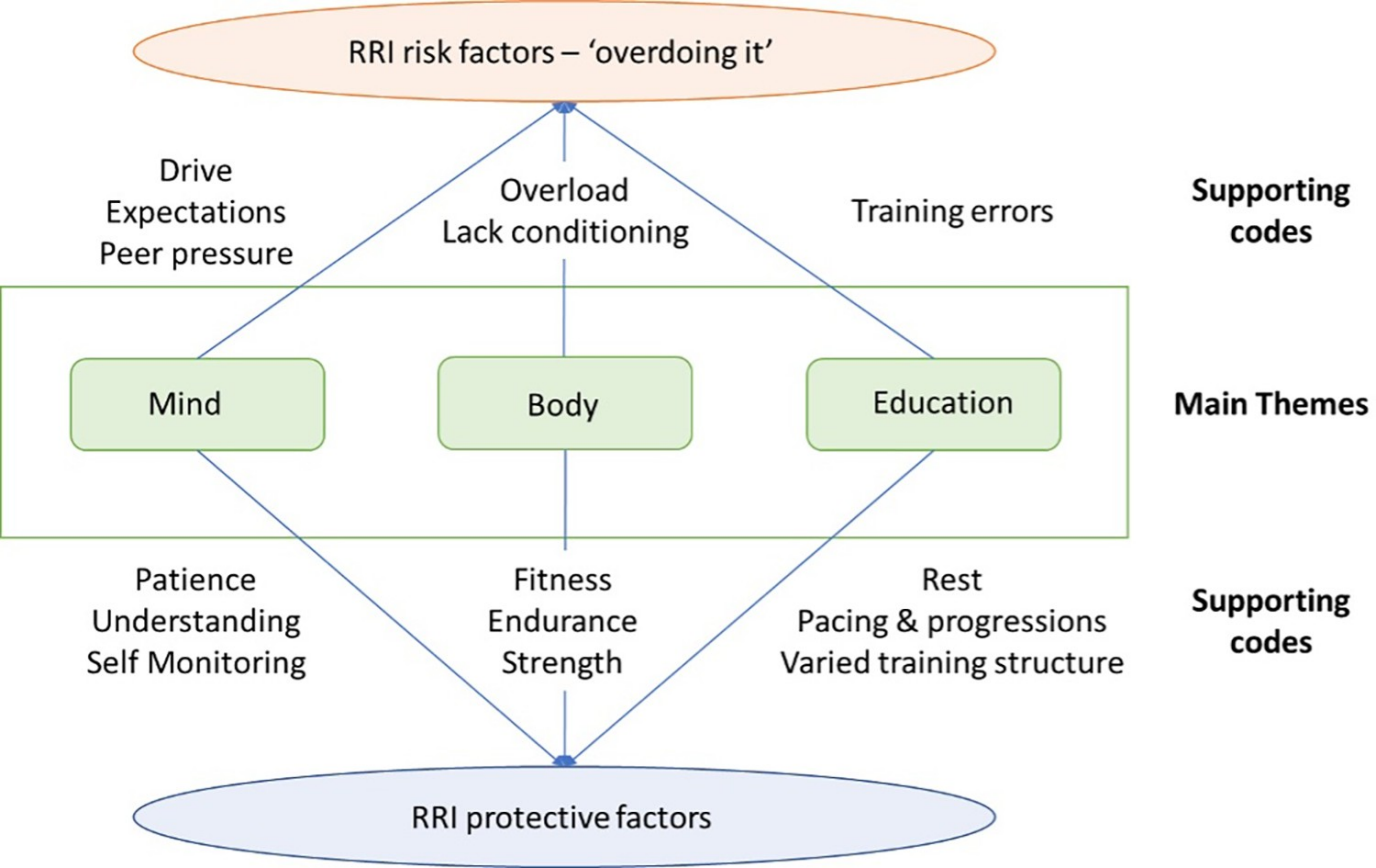
The internal locus of injury.

Following this analysis, three main themes relating to recreational runner’s views regarding RRI causation and prevention were identified: Mind, Body and Education. The first theme, ‘Mind’, describes the runners’ views that a level of self-understanding and self-management of their current physical state is required to avoid RRI. The second theme, ‘Body’, reflects their opinions that injury free running requires a degree of physical fitness and strength. Finally, the third theme, ‘Education’, represents their beliefs that specific coaching or training in appropriate running and training loads can assist in avoiding RRI. The three main themes form separate pillars of what we describe as an ‘internal locus of injury’ model (Fig 1).

When asked what they thought were the biggest contributors to RRI, and if there were ways in which RRI could be prevented, the participants first responses revolved around the concept of ‘overdoing it’.

“I think it is progression of loading. I think if you load up too much, too quickly that’s primarily when people get injured.” *Cain*

“probably going too hard too early.” *Kyle*

“Definitely overtraining. Too hard, too often.” *Tim*

“Overtraining and too much too soon, yeah just overloading.” *Jon*

As described by the runners, there were multiple components contributing to them ‘overdoing it’–which we represent as three themes: Mind, Body and Education. Acting both alone and in combination, imbalances occurring within these themes were seen to contribute to ‘overdoing it’ and therefore to be involved in both RRI causation and prevention. The three themes are described in further detail below.

### Mind theme

Regarding the Mind theme, the participants believed that an individual’s approach to running and the decisions they make during training could lead to RRI. They describe a certain degree of self-understanding and self-management required for RRI prevention, and alternately how individual drivers can contribute to RRI causation. Firstly, they believe an *Understanding* of how their body is coping with the current training load is crucial.

“It’s just knowing where you’re at.” *Pete*

“Having a little bit of knowledge and a little bit of understanding of what your body can and can’t do.” *Jon*

“I think the trick is making sure when you are doing your intervals that you are going at your own pace.” *Cain*

“That’s one of the biggest things, monitoring yourself and your training. You’re the only one who knows how your body feels.” *Jon*

“as an experienced runner, you know when you’re running hard, and when you’re running easy, and when you might be on the borderline of an injury or something like that.” *Tim*

In conjunction with self-understanding the runners then consider that *Self-monitoring and Patience* is also required to avoid RRI.

“patience is the biggest thing. Patience and consistency, but they’re kind of the hardest things as well.” *Jon*

“the coach won’t be there all the time and yeah you can do a bit of your self-managing.” *Flynn*

“So, education for novice runners but …. maybe self-control for more experienced runners?” *Tim*

“taking a little bit longer to get started. I think that’s a good way of doing it.” *Cain*

The runners are also aware that despite their best intentions and self-understanding, certain internal (*Drive*, *Expectations*) and external (*Peer pressure*) factors can disrupt their self-management and contribute to RRI.

“So, it might even be people’s expectations as opposed to their fitness level.” *Pete*

“if you are not injured, or haven’t been injured for a while, you don’t really pay attention.” *Kyle*

“a lot of the watches have the record, so you are trying to beat your record.” *Kyle*

“when you’re training hard, and you’ve got goals in mind, I think performance probably comes before injury prevention” *Jon*

“I reckon peer pressure comes into it sometimes.” *Cain*

### Body theme

The Body theme refers to a certain level of physical conditioning (*Fitness*, *Endurance*, *Strength*) that was deemed by the runners to be a critical component in avoiding ‘overdoing it’, which could then result in RRI. Also, on the opposite side, that a *Lack of conditioning or Overload*, could contribute to ‘overdoing it’ and RRI.

“just having the muscle endurance to be able to run a longer distance.” *Cain*

“All our running is really up to us and our strength and our fitness.” *Pete*

“postural stuff too. There’s lots of upper body strength that you need for running.” *Pete*

“I reckon the main thing is your conditioning, whether you are strong enough, whether you have enough muscle endurance, as well as your ability to carry your weight.” *Cain*

“like if you just go and run stairs after not doing any kind of you know hill work or base aerobic work.” *Tim*

“Like a week off and you feel it. You think I can’t do what I did last week, and it was only a week.” *Kyle*

### Education theme

Finally, the runners considered that an Education component, some degree of instruction or coaching in how to structure a running program (*Rest*, *Pacing and progressions*, *Varied training structure*), was a necessary part of the process of avoiding RRI, and that lack of knowledge (*Training errors*) could result in ‘overdoing it’.

“With recreational runners I think it is just that they are uneducated on how to structure their training.” *Tim*

“like there’s so many online programs and coaches and things going on. I think that’s a great thing.” *Jon*

“Couch to 5k. They’ve done a really good job and they haven’t got injuries along the way.”

“I found coaching to be pretty important, more capable of running faster without necessarily having injuries.” *Cain*

“Apps—It is just another way of measuring how to do things. Making sure you are not overdoing things.” *Pete*

Two additional factors raised by the participants, running footwear and running environment, are also considered in the Education theme. While the participants believed there were involved in RRI, there were divergent opinions regarding whether these factors were causative or protective. This is a reflection of the evidence presented in the Introduction section, that runners’ beliefs regarding RRI and prevention are often not based on the evidence.

Several runners believed that the age and the design of a shoe could be an important contributor to RRI.

“I definitely feel that it is important to update the shoe regularly though. I was getting foot pain, running in a pair 6 years old.” Pete

“I totally agree with that in relation to the shoes—an old pair of shoes.” Kyle

“You buy shoes for the cushioning and the padding—as a protective device.” Pete

Whereas other runners either dismiss the idea that shoes play a role in the development of RRI or believe that if the shoe does contribute to RRI it is because the wrong style of footwear has been used for training or running.

“footwear—I think people think it plays more of a factor than it does.” Tim

“I guess it could be wearing the wrong pair of shoes too—racing flats for long runs.” Flynn

Similarly, different running environments were seen to be a causative factor for RRI by some runners.

“geography or topography can have a bit to do with injury. Running in the bush.” Cain

“And totally if you are not used to running with hills and suddenly you start running hills it’s a completely different style of running,” Kyle

“Like going from your cross country–winter stuff–to having your first track session.” Flynn

While other runners viewed varied environments as a protective factor for RRI.

“I think that surfaces play a role as well. Like just getting a mix of everything, so bush or dirt or trail and grass.” Jon

“Road is not as bad as everyone thinks–you need it to kind of harden your muscles in your legs up.” Jon

### Internal locus of injury model

Considered together, these three themes can be seen to form an ‘internal locus of injury’ model for RRI prevention and causation (Fig 1). Each theme by itself, when not well managed or well understood by the runners, is capable of upsetting the balance, leading to ‘overdoing it’, and contributing to RRI. Conversely, the runners describe a model of how they believe they can act to minimise the chances of RRI—by maintaining their physical fitness, monitoring their motivations and their bodies response to training, and educating themselves regarding appropriate running and training loads.

The runners’ perception of having a high degree of personal control regarding prevention of RRI, ‘the internal locus of injury’, also extends to their management of RRI. When asked if, and what sort of help, they had sought after experiencing RRI, they mostly described initially self-managing.

“I self-managed, I guess. I eased back on training.” *Pete*

“once I got through the acute phase of it, it was trying to do some stretching and then

strengthening.” *Cain*

“just talking to people. I was overseas once and got a bit of a niggle and just talked to a few different people, got some advice, got some exercises and you know managed myself.” *Jon*

“I guess sometimes self-managing an injury, if I’m jogging, I’ll really focus on running on the grass just to lessen the impact, or even just warming-up on grass.” *Flynn*

Although some of the runners did report seeking professional help if the initial self-management didn’t work, or if the injury was more severe.

“I’ve had torn patella tendon, that was a big one that took a couple of years to heal. Definitely saw a healthcare professional–a physio.” *Kyle*

“I’ve had a couple that were a bit worse, and I’ve got a mate who’s a physio so I’ve been treated by him a few times in the past.” *Jon*

“I try to self-manage but I have definitely gone and seen physios and podiatrists.” *Tim*

## Discussion

This study used reflexive thematic analysis to investigate the perceptions and views of male recreational runners in regard to their understanding of RRI causation, prevention and management. Three main themes were identified—‘Mind’, ‘Body’ and ‘Education’, which combined describe an “internal locus of injury” model. This model depicts the runners’ viewpoint that RRI results from ‘overdoing it’ and that prevention of RRI requires management of both their own personal physical and mental capabilities. The interpretative phenomenological analysis method used allows for a broader perspective of the runner and RRI to be examined, one which incorporates psychological and social factors, as well as the more widely researched biomedical, biomechanical and physiological factors.

When it comes to identifying factors implicated in the development of RRI, these runners name ‘overdoing it’ as a main contributor, which concurs with current academic literature [3, 8]. Higher training loads, including longer weekly running distance, longer duration of running or higher frequency of running, have all been shown to contribute to RRI [3, 8]. This factor, overloading, has also been identified in other qualitative studies investigating runners’ perceptions of the aetiology of RRI [12, 14]. The runners also believe that having a better understanding of how to structure a running program is critical to avoiding ‘overdoing it’ and RRI. This could take the form of personalised coaching or alternatively could just be a generic online exercise program (e.g. ‘Couch to 5k’ [21]) or app. Again, there is research evidence that supports their beliefs that coaching or education can reduce rates of RRI [22, 23]. Novice runners displayed lower rates of RRI when using a structured program (Couch to 5k) than when using a self-devised program [23], and ongoing online personalised advice also resulted in a lower incidence of RRI compared to one session of RRI advice in a group of Dutch trail runners [22].

Sports research has traditionally defined risk factors as intrinsic (athlete related factors) or extrinsic (environmental risk factors) which does not necessarily reflect the often multifactorial nature of RRI and provides limited insight into possible interventions to reduce injury [24]. Alternatively, injury related risk factors can be described as non-modifiable or modifiable [24]. Modifiable risk factors are especially important when developing injury prevention programs as they can be targeted by physical training (strength, balance or flexibility), behavioural or educational interventions [24, 25]. Interestingly the themes and supporting codes generated in this analysis (Mind, Body, Education), that the runners describe as contributing to ‘overdoing it’, could all be described as modifiable risk factors. Verhagen et al. [14] also identified the role modifiable risk factors play in RRI prevention. These authors describe self-regulation as the main process by which runners deal with complaints and injuries and note that self-regulation of running activity is influenced by competition schedules, performance goals and individual drive. New strategies targeting modifiable risk factors are being developed to improve injury-preventive behaviour in runners, although as yet there is no evidence of their effectiveness [25]. In addition to being modifiable, the themes and codes were identified by analysis of the runners’ own voices and perspectives. The lack of success of past interventions and investigations in reducing rates of RRI may be in part because the largely quantitative methods used did not consider the runners’ perspective and needs. The key to improving the current low compliance to injury prevention programs may lie in the inclusion of the runners’ perspectives in the development of future programs [15].

### Clinical implications

Prior research has identified that recreational runners utilise personal experience, peer group, anecdotal or web-based advice or information received from running stores, in preference to professional advice or research evidence [12, 14, 26, 27]. This was reflected in the results of this analysis, where the participants preference for self-management was obvious both in their approach to RRI prevention and also to management of any RRI they experienced. Self-management, informed by a mix of personal experience and information gained online, from peers and experts, can be a valid treatment and prevention option for runners and has been reported previously [14]. However, this preference could have adverse consequences if it results in runners seeking professional health advice late, or incorrectly self-diagnosing an injury, or following incorrect treatment advice from blogs or untrained staff [12, 26, 27]. It may be necessary for health care professionals (HCP) hoping to assist in reducing RRI, to recognise that runners desire autonomy when making decisions regarding their running situation, and to support them in making educated decisions [14]. This is obviously problematic if HCPs are not a first line resource for runners, and so alternate approaches may be required.

One pathway for HCPs to get involved may be by assisting runners to improve their RRI knowledge (the Education theme of the model). As runners have been reported to mostly search online for information, this may take the form of the provision of information through websites or blogs regarding RRI symptoms, treatment, and alternative activities to help runners deal with injuries on their own [14, 27]. A further connection with runners may be established if the HCPs emphasise their practical running experience on these channels, in addition to their academic credentials [26]. Additionally, the HCP could consider how they may support the runners in their development to ‘experienced runners’. One study has shown that runners with less than six months experience are 1.5 times more likely to be injured as those with 2–5 years of running experience, and almost two times more likely to be injured than those with 5–10 years of running experience [23]. The authors suggested this may be a result of the experienced runners having a better understanding of their personal injury threshold and a method of managing it. As one of the runners in our study stated, ‘as an experienced runner, you know when you’re running hard, and when you’re running easy, and when you might be on the borderline of an injury or something like that’. While the Mind and Body themes in our suggested ‘internal locus of injury’ model may only develop with time and distance covered, HCPs can assist runners build skills in the Education theme. This may take the form of provision of links to evidence-based resources, such as Couch to 5k, that assist runners in developing consistency, sensible progressions and rest days in their running schedule.

### Limitations

The findings of this study should be considered in light of certain limitations. This study only included male runners, so the findings are not representative of female running populations. In addition, the runners were experienced recreational runners and so the findings may not be transferable to other running populations such as novice runners. While some sampling and self-selection bias may be expected with the use of a snowball sampling technique, it is commonly used in qualitative research where the results are not assessed using traditional statistical approaches [28]. Although the sample size of this study is relatively small (six), the reflexive thematic analysis approach used by Braun and Clarke [29] does not specify a particular sample size or saturation of data, only that the sample size allows for generation of themes and complex analysis. One of the focus group sessions took place online via Zoom^TM^ and personal interactions in this session may have been affected by the physically distanced process.

## Conclusion

This study has provided a deeper understanding of how Australian male recreational runners view RRI causation, prevention, and management. The findings of this study identified that recreational runners rely on self-management of their fitness and training, in preference to professional or academic advice, to avoid and manage RRI. Health care professionals may be able to use online resources to assist runners to develop appropriate running training knowledge and reduce the likelihood of developing a RRI, as this is one of the runners’ preferred information sources.

The modifiable RRI risk factors identified in this study, through examination of the runners’ perspectives and experiences, could be considered when designing future RRI prevention programs. Consideration of the runners’ viewpoints during development of RRI prevention programs may provide more opportunities for improving compliance to the programs and reducing future rates of RRI.

## Supporting information

S1 File Interview questions. (DOCX)

S2 File Steps undertaken to complete the reflexive thematic analysis as described by Braun and Clarke [19, 20]. (DOCX)
