# Supplementary material for: Going their own way–male recreational runners and running-related injuries: A qualitative thematic analysis
Source: PLoS One. 2022 Aug 25;17(8):e0273401. doi: 10.1371/journal.pone.0273401 (PMC9409521; doi:10.1371/journal.pone.0273401)
Supplement: S1 File — (DOCX) [file pone.0273401.s001.docx]

**Supporting information file 1**

**Focus group Interview questions**

1) What do you think are the biggest contributors to running-related injuries?

2) Are there any ways in which you think injuries can be prevented??

3) For those of you who have ever experienced a running-related injury, how did you go about managing that injury?

4) Where do you buy your running shoes? What makes you decide to buy new ones I am interested in where you buy them, for example in-store, or online, and why; as well as what influences you to select a particular shoe for your running.

5) What factors influence which type of running shoe you buy?
