# Supplementary material for: Going their own way–male recreational runners and running-related injuries: A qualitative thematic analysis
Source: PLoS One. 2022 Aug 25;17(8):e0273401. doi: 10.1371/journal.pone.0273401 (PMC9409521; doi:10.1371/journal.pone.0273401)
Supplement: S2 File — (DOCX) [file pone.0273401.s002.docx]

**Supporting Information File 2**

**Steps undertaken to complete the reflexive thematic analysis as described by Braun and Clarke (2006 and 2019)**

Braun and Clarke (2006) initially described 6 phases of thematic analysis, and provided further clarity on generation of themes Braun and Clarke (2019)

1. Familiarizing yourself with your data: transcribing, reading and re-reading the data, noting down initial ideas
2. Generating initial codes: coding interesting features of the data in a systematic fashion across the entire data set, collating data relevant to each code
   1. Quality reflexive TA is not about following procedures ‘correctly’ (or about ‘accurate’ and ‘reliable’ coding, or achieving consensus between coders), but about the researcher’s reflective and thoughtful engagement with their data and their reflexive and thoughtful engagement with the analytic process.
   2. If more than one researcher is involved in the analytic process, the coding approach is collaborative and reflexive, designed to develop a richer more nuanced reading of the data, rather than seeking a consensus on meaning
3. Generating themes (initially Searching for themes in 2006):
   1. Changed to emphasise that themes are not ‘in’ the data, pre-existing analysis, awaiting retrieval
   2. Themes do not passively emerge from either data or coding
   3. Themes are creative and interpretive stories about the data, produced at the intersection of the researcher’s theoretical assumptions, their analytic resources and skill, and the data themselves.
4. Reviewing themes: checking if the themes work in relation to the coded extracts and the entire dataset
5. Defining and naming the themes: ongoing analysis to refine the specifics of each theme, generating clear definitions and names for each theme
6. Producing the report: selection of vivid, compelling extract examples relating back to the analysis of the research question and literature, producing a scholarly report of the analysis
